# Genotyping of *Aspergillus fumigatus* in Formalin-Fixed Paraffin-Embedded Tissues and Serum Samples From Patients With Invasive Aspergillosis

**DOI:** 10.3389/fcimb.2018.00377

**Published:** 2018-10-23

**Authors:** Theun de Groot, Ferry Hagen, Willem Vreuls, Paul E. Verweij, Anuradha Chowdhary, Jacques F. Meis

**Affiliations:** ^1^Department of Medical Microbiology and Infectious Diseases, Canisius Wilhelmina Hospital, Nijmegen, Netherlands; ^2^Department of Medical Mycology, Westerdijk Fungal Biodiversity Institute, Utrecht, Netherlands; ^3^Department of Clinical Pathology, Canisius Wilhelmina Hospital, Nijmegen, Netherlands; ^4^Centre of Expertise in Mycology Radboudumc/CWZ, Nijmegen, Netherlands; ^5^Department of Medical Microbiology, Radboudumc, Nijmegen1, Netherlands; ^6^Department of Medical Mycology, Vallabhbhai Patel Chest Institute, University of Delhi, New Delhi, India

**Keywords:** *Aspergillus fumigatus*, molecular detection, molecular typing, paraffin embedded formalin fixed tissue, serum

## Abstract

Invasive aspergillosis (IA) is a deep tissue infection with a high mortality occurring mostly in immunocompromised patients. To investigate the pathology of patients with IA it may be important to determine the genotype of the invasive isolate of *Aspergillus*, however available tissues for study are often formalin fixed paraffin embedded (FFPE). Although DNA has been successfully isolated from such tissues for species identification, genotyping of *Aspergillus* species on such tissues has not yet been performed. In this study we aimed to determine the genotype of *Aspergillus fumigatus* in FFPE tissue and serum samples from five patients with invasive aspergillosis using nine highly polymorphic short tandem repeat (STR*Af*) loci. FFPE lung and bronchial biopsies from all patients were successfully typed. By comparing the latter result with non-FFPE materials from non-sterile samples such as sputum, bronchoalveolar lavage and lung abscess, we found identical genotypes within three patients, while the two other patients had a dominant genotype shared among all sample types. Genotyping of serum samples was successful in two serum samples with galactomannan ratios of 4 and 5.6, but failed in serum samples with galactomannan levels <0.5. In addition, testing a subset of these materials with the AsperGenius multiplex qPCR assay, we did not find azole resistance mutations. With this STR*Af* assay, *A. fumigatus* from FFPE tissue and serum was successfully genotyped, allowing retrospective examination of *A. fumigatus* in culture negative patients with IA.

## Introduction

The genus *Aspergillus* consists of more than 300 species of spore-forming filamentous fungi, but only a few species are recognized as human pathogens (Paulussen et al., 2017). While healthy individuals are generally not affected, immunocompromised persons are at risk to develop invasive aspergillosis (IA) with high mortality rates (Ullmann et al., 2018). *Aspergillus fumigatus* is the most prevalent species within the genus *Aspergillus* and the major species causing this disease, although there are also other *Aspergillus* species, which can cause IA (Sugui et al., 2014).

The diagnosis of IA is challenging as its clinical symptoms, including fever, cough, respiratory secretions, and dyspnea are not specific for this disease. Readily available respiratory samples, like sputum and bronchoalveolar lavage (BAL) fluid, can be cultured or directly tested for the presence of *Aspergillus* species, however positive specimens may reflect environmental contamination or colonization of the airway instead of invasive infection (Rantakokko-Jalava et al., 2003; Springer et al., 2018b). To improve the quality of clinical studies, guidelines have been established by the European Organization for Research and Treatment of Cancer/Mycosis Study Group (EORTC/MSG) to diagnose IA by categorizing this invasive fungal disease in “proven,” “probable,” or “possible” (De Pauw et al., 2008). Proven IA requires the histopathological identification of hyphae in a needle aspiration or biopsy accompanied by tissue damage and culture from a tissue that is normally sterile, followed by identification of the *Aspergillus*. Probable IA is based on a combination of host factors, clinical features and mycological criteria, while with possible IA these mycological criteria are not required. Mycological criteria include the identification of *Aspergillus* species in respiratory samples or their cultures or the presence of galactomannan (GM) antigen, a component of the *Aspergillus* cell wall (Mennink-Kersten et al., 2004; Boch et al., 2016; Eigl et al., 2017), in serum, plasma, or BAL fluid (De Pauw et al., 2008). Due to difficulties to use these diagnostic criteria for intensive care patients, an additional classification of “putative” IA was established, which besides a positive culture of lower respiratory tract specimen, is based on clinical signs and host factors or mycological criteria (Blot et al., 2012).

Different *Aspergillus* species have variable susceptibility for antifungal drugs and therefore it is important to identify the species causing IA, preferentially using deep tissue biopsies to be certain that the fungus recovered is causing the infection. For histopathological purposes, such tissues are mostly fixed with formalin and embedded in paraffin (Ullmann et al., 2018). Microscopic examination is sometimes challenging, especially for *Aspergillus* species, leading quite frequently to an incorrect morphological diagnosis (Kung et al., 2018). Recent advances allowed the isolation of fungal DNA from formalin-fixed paraffin-embedded (FFPE) tissues and, using qPCR, the identification of the fungal pathogens to species level (O'Sullivan et al., 2003; Rantakokko-Jalava et al., 2003; Perlin and Wiederhold, 2017). This has also been shown for samples with *Aspergillus* (Salehi et al., 2016; Dannaoui et al., 2017; Dudakova et al., 2017; Barnes et al., 2018; Morio et al., 2018; Springer et al., 2018a). To obtain closer insight in the epidemiology and pathogenesis of IA, it is essential to characterize *Aspergillus* strains genetically from different tissues from patients with IA. Different high discriminatory typing techniques for *A. fumigatus* are available (de Valk et al., 2007). As human DNA is also present in tissue and serum samples, such genotyping assay should be species-specific, making AFLP (de Valk et al., 2007) and RISC (de Ruiter et al., 2007) typing unsuitable. In contrast, microsatellite typing using the STR*Af* assay only amplifies specific *A. fumigatus* targets and the method is highly discriminative (de Valk et al., 2005). In this study we investigated whether it is possible to genotype *A. fumigatus* with high-resolution STR*Af* typing (de Valk et al., 2005) in FFPE tissue and serum samples.

## Materials and methods

### Patients

Isolates, FFPE tissue and serum samples from five ICU patients (A-E) in the age of 58 to 67 years old, were collected. All five patients had histological proven IA and were treated with antifungals. An overview of the patients is given in Table 1.

**Table 1:** Patients with histologically proven invasive aspergillosis.

**Patient**	**Age**	**Underlying disease**	**Diagnosis**	**Treatment**	**Outcome**
A	67	COPD, *Influenza* pneumonia (ICU)	Pulmonary IA	Itraconazole caspofungin	Died
B	62	COPD, *Legionella* pneumonia (ICU)	Disseminated IA	Itraconazole caspofungin	Died
C	59	COPD, Lung carcinoma (ICU)	Pulmonary IA	Caspofungin	Died
D	58	COPD	Subacute IA	Itraconazole	Survived
E	65	COPD	Pulmonary IA	Itraconazole	Survived

### DNA extraction from serum and FFPE tissue samples

DNA was extracted from 200 μl serum using the High Viral Nucleic Acid Kit (Roche Diagnostics, Almere, The Netherlands) according to manufacturer instructions. For the FFPE lung and bronchial biopsies, 5 sections (thickness, 10 μM) were cut using a sterile microtome blade and transferred to a 2 ml centrifuge tube. To remove paraffin wax, the samples were washed with 2x 500 μl xylene, 2x 500 μl ethanol absolute and 500 μl acetone. The pellet was dried at 56°C. Next, 300 μl of Puregene cell lysis solution from Gentra Systems (Biozym B.V., Landgraaf, The Netherlands) and Proteïnase K was added and incubated overnight at 56°C with continue mixing. Following mechanical lysis by running the sample for 30 s at 6,500 rpm in a MagNA Lyser instrument with MagNA Lyser Green Beads (Roche Diagnostics), DNA was purified using affinity chromatography and subsequently eluted with 10 mM Tris-HCl, 1 mM EDTA (Boom et al., 1990).

### *A. fumigatus* isolates and DNA extraction

Samples obtained from patients were routinely cultured on Sabouraud agar for 7 days at 30 and 35°C. *A. fumigatus* isolates were presumptively identified at the time of collection by macroscopic and microscopic characteristics and the ability to grow at 48°C. The isolates were stored as spore suspensions in regular microbial freezing broth containing 12.5% (vol/vol) glycerol at −80°C until testing. The isolates were revived by scraping off part of the sample, plating on Sabouraud's agar and cultivated at 30°C until sporulation. DNA from *A. fumigatus* spores was extracted and purified with the MagNA Lyzer and MagNA Pure LC Instruments (Roche Diagnostics) as previous described (de Valk et al., 2005).

### Genotyping using the STR*Af* assay

Reaction conditions and PCR primers were as described (de Valk et al., 2005), with minor modifications as described below. With regard to the serum and tissue samples the volume of DNA was raised to 5 μl for each sample, the total volume of the PCR reaction remained 25 μl. The number of cycles was increased to 40 during the amplification procedure. Electropherograms were analyzed using Fragment Profiler 1.2 software (GE Healthcare, Roosendaal, The Netherlands).

### Aspergenius multiplex qPCR

The AsperGenius multiplex qPCR assay (PathoNostics, Maastricht, the Netherlands) was used to detect *Aspergillus* species, *A. fumigatus* and *A. terreus*, and identify azole resistance *CYP51A* mutations TR_34_, L98H, Y121F and T289A in *A. fumigatus* according to the instructions from the manufacturer and as described (Chong et al., 2015). The qPCR was performed on a LightCycler 480-II (Roche Diagnostics).

### Ethics

Samples were collected during routine patient care and the study was retrospective, therefore it was determined by the local Institutional Review Board of the CWZ that ethical clearance was not indicated. As patient anonymity was maintained, written informed consent of participants was also not required.

## Results

From patient A, BAL, lung abscess aspirate and four sputa, collected within a week, were cultured and genotyped by analyzing the variability in length of 9 STRs in *A. fumigatus* (STR*Af*) The BAL and four sputa showed one identical genotype of *A. fumigatus*, while a different genotype was obtained from the lung abscess (Table 2). Additionally, two sera and three lung biopsies were obtained. The serum samples, which were GM negative, showed no signal with the STR*Af* assay. In two lung biopsies different genotypes were identified, as multiple peaks were present for all 9 STR*Af* markers. The genotype of the third lung biopsy was identical to the one found in the BAL and sputum. From patient B we genotyped *A. fumigatus* isolates from 13 sputa, an ascites fluid and a BAL. One *A. fumigatus* STR*Af* type was found for all cultures and this type was also detected in two serum samples, with GM ratios of 4.0 and 5.6, and FFPE lung tissue. STR*Af* typing was not successful for a serum sample with a GM index of 0.4.

**Table 2:** Overview of typing results.

**Pat. + no**.	**Origin**	**Day of isolation**	**Cultured isolate**	**STR*****Af*** **type**								
				**2A**	**2B**	**2C**	**3A**	**3B**	**3C**	**4A**	**4B**	**4C**
A1	Sputum	0	Yes	25	22	19	28	9	27	10	9	5
A2	Sputum	2	Yes	25	22	19	28	9	27	10	9	5
A3	Serum, GM 0.1	3	No	neg	neg	neg	neg	neg	neg	neg	neg	neg
A4	Serum, GM 0.1	6	No	neg	neg	neg	neg	neg	neg	neg	neg	neg
A5	Sputum	7	Yes	25	22	19	28	9	27	10	9	5
A6	Lung abscess	7	Yes	23	21	8	46	9	6	8	9	5
A7	Sputum	7	Yes	25	22	19	28	9	27	10	9	5
A8	Biopsy lung	7	No	> 3 genotypes^*^			> 3 genotypes^*^			> 3 genotypes^*^		
A9	BAL	9	Yes	25	22	19	28	9	27	10	9	5
A10	Biopsy lung	9	No	> 3 genotypes^*^			> 3 genotypes^*^			> 3 genotypes^*^		
A11	Biopsy lung	9	No	neg	22	19	28	9	27	10	9	5
B1	Sputum	0	Yes	18	12	8	28	10	18	9	8	5
B2	Sputum	1	Yes	18	12	8	28	10	18	9	8	5
B3	Sputum	3	Yes	18	12	8	28	10	18	9	8	5
B4	Sputum	7	Yes	18	12	8	28	10	18	9	8	5
B5	Sputum	10	Yes	18	12	8	28	10	18	9	8	5
B6	Sputum	14	Yes	18	12	8	28	10	18	9	8	5
B7	Sputum	16	Yes	18	12	8	28	10	18	9	8	5
B8	Sputum	20	Yes	18	12	8	28	10	18	9	8	5
B9	Sputum	30	Yes	18	12	8	28	10	18	9	8	5
B10	Serum, GM 0.4	30	No	neg	neg	neg	neg	neg	neg	neg	neg	neg
B11	Sputum	31	Yes	18	12	8	28	10	18	9	8	5
B12	Sputum	35	Yes	18	12	8	28	10	18	9	8	5
B13	Sputum	38	Yes	18	12	8	28	10	18	9	8	5
B14	BAL	42	Yes	18	12	8	28	10	18	9	8	5
B15	Ascitis fluid	42	Yes	18	12	8	28	10	18	9	8	5
B16	Serum, GM 4.0	43	No	18	12	8	28	10	18	9	8	5
B17	Sputum	43	Yes	18	12	8	28	10	18	9	8	5
B18	Serum, GM 5.6	44	No	18	12	8	28	10	18	9	8	5
B19	Biopsy lung	44	No	18	12	8	28	10	18	9	8	5
C1	Sputum	0	Yes	21	25	18	27	12	7	21	10	8
C2	Sputum	0	Yes	18	20	15	28	11	21	26	26	8
C3	Bronchial biopsy	1	No	neg	neg	18	27	12	7	neg	10	8
C4	Serum	1	No	neg	neg	neg	neg	neg	neg	neg	neg	neg
C5	Sputum	4	Yes	18	28	15	16	10	19	8	8	5
C6	Sputum	5	Yes	21	25	18	27	12	7	21	10	8
C7	Sputum	21	Yes	21	25	18	27	12	7	21	10	8
C8	Sputum	25	Yes	21	25	18	27	12	7	21	10	8
C9	Serum	25	No	neg	neg	neg	neg	neg	neg	neg	neg	neg
C10	Sputum	32	Yes	21	25	18	27	12	7	21	10	8
C11	Sputum	39	Yes	21	25	18	27	12	7	21	10	8
C12	Sputum	42	Yes	21	25	18	27	12	7	21	10	8
C13	Sputum	45	Yes	21	25	18	27	12	7	21	10	8
C14	Sputum	46	Yes	21	25	18	27	12	7	21	10	8
C15	Sputum	49	Yes	21	25	18	27	12	7	21	10	8
C16	Sputum	54	Yes	21	25	18	27	12	7	21	10	8
C17	Sputum	61	Yes	21	25	18	27	12	7	21	10	8
C18	Sputum	63	Yes	21	25	18	27	12	7	21	10	8
C19	Sputum	68	Yes	21	25	18	27	12	7	21	10	8
C20	Sputum	71	Yes	21	25	18	27	12	7	21	10	8
C21	Sputum	75	Yes	21	25	18	27	12	7	21	10	8
C22	Sputum	75	Yes	18	12	18	27	22	21	18	9	5
C23	Serum	76	No	neg	neg	neg	neg	neg	neg	neg	neg	neg
C24	Sputum	76	Yes	21	25	18	27	12	7	21	10	8
C25	Serum	77	No	neg	neg	neg	neg	neg	neg	neg	neg	neg
C26	Sputum	79	Yes	21	25	18	27	12	7	21	10	8
C27	Sputum	79	Yes	21	25	18	27	12	7	21	10	8
C28	Bronchial biopsy	79	No	21	25	18	27	12	7	21	10	8
D1	Sputum	0	Yes	11	12	17	26	21	21	14	8	5
D2	BAL	1	Yes	11	12	17	26	21	21	14	8	5
D3	BAL	1	Yes	11	12	17	26	21	21	14	8	5
D4	Biopsy lung^#^	3	Yes	11	12	17	26	21	21	14	8	5
D5	Lung abscess	3	Yes	11	12	17	26	21	21	14	8	5
D6	Serum	4	No	neg	neg	neg	neg	neg	neg	neg	neg	neg
D7	Serum, GM 0.2	4	No	neg	neg	neg	neg	neg	neg	neg	neg	neg
D8	Biopsy lung	4	No	11	12	17	26	21	21	14	8	5
E1	BAL	0	Yes	18	23	16	38	11	46	10	9	8
E2	Bronchial biopsy	0	No	18	23	16	38	11	46	10	9	8

GM, galactomannan.

FFPE tissues are underlined.

* Genotypes from these samples differed from the genotypes found in the other samples.

# *Genotyping was performed on both culture as direct material. Outcome was identical*.

From patient C, cultures of 22 sputum samples, isolated during a 9-week period, were STR*Af* typed. Nineteen of these *A. fumigatus* isolates were genotypically identical, while three isolates demonstrated three different genotypes. Two bronchial biopsies demonstrated a STR*Af* type identical to the 19 sputum cultures. Four GM negative serum samples of this patient were also negative in the STR*Af* assay. In patient D STR*Af* typing of five cultured *A. fumigatus* isolates from sputum, two BAL samples, lung tissue and a lung abscess yielded a genotype that was identical to the genotype obtained from FFPE lung tissue. Two serum samples from this patient were negative for STR*Af* typing. In patient E the STR*Af* type found in the culture from a BAL sample was identical to the one obtained from a FFPE bronchial biopsy (Table 2).

Finally, we investigated whether it was feasible to determine azole resistance in a subset of available FFPE tissue and serum samples using the AsperGenius multiplex qPCR assay. This assays allows detection of *Aspergillus* in clinical materials and subsequent identification of azole resistance mutations TR_34_, L98H, Y121F, and T298A in *CYP51A* in case of *A. fumigatus* positive sampes. From 10 samples with successful STR*Af* typing, 9 were found positive for both the *Aspergillus* species and *A. fumigatus* qPCR (Table 3). From 7 sera with unsuccessful STR*Af* typing (A3, A4, B10, C4, C9, C25, and D7), *A. fumigatus* was identified in 4 samples using the AsperGenius qPCR. There was no azole-resistance related mutation observed in the *A. fumigatus* positive samples.

**Table 3:** Results of AsperGenius multiplex real-time PCR assay on subset of patient materials.

**Patient + No**.	**Origin**	**Ct** ***Aspergillus* species**	**Ct** ***A. fumigatus***	**Ct** ***A. terreus***	***A. fumigatus CYP51A*** **PCR**			
					**WT/TR_34_**	**WT/L98H**	**WT/Y121F**	**WT/T289A**
A3	Serum, GM 0.1	35.49	38.60	–	-	—	WT	WT
A4	Serum, GM 0.1	35.49	36.93	–	–	WT	WT	WT
A8	Biopsy lung	26.87	28.61	–	WT	WT	WT	WT
A10	Biopsy lung	30.80	32.55	–	WT	WT	WT	WT
A11	Biopsy lung	31.61	33.43	–	WT	WT	WT	WT
B10	Serum, GM 0.4	31.83	32.44	–	–	WT	WT	–
B16	Serum, GM 4.0	29.78	31.39	–	#	WT	WT	WT
B18	Serum, GM 5.6	32.41	33.93	–	WT	WT	WT	WT
B19	Biopsy lung	–	–	–			
C3	Bronchial biopsy	32.55	34.36	–	WT	WT	-	-
C4	Serum	–	–	–			
C9	Serum	–	–	–			
C25	Serum	36.41	38.56	–	WT	WT	WT	WT
C28	Bronchial biopsy	26.76	28.78	–	WT	WT	WT	WT
D7	Serum, GM 0.2	–	–	–			
D8	Biopsy lung	30.70	32.45	–	WT	WT	WT	WT
E2	Bronchial biopsy	30.77	32.62	–	WT	WT	WT	WT

–, Amplification not successful.

*# Melting curve differ*.

## Discussion

This study demonstrated that it is technically feasible to genotype *A. fumigatus* directly from FFPE tissue samples from patients with proven IA without the need to cultivate the isolate. In three patients (B, D, and E) we observed identical STR*Af* types in all sample types, including sputa and BAL fluids, serum, and lung biopsies. In patient C a dominant genotype was found in cultures from sputa, which was also identified in lung tissue. In patient A, five of the six isolates had the same genotype as detected in one of the three lung biopsies. Remarkably, in the two other lung biopsies more than three STR*Af* types were found, suggesting mixed infection, a phenomenon described earlier (Kolwijck et al., 2016). Interestingly, another study found that from such mixed respiratory infections, only one strain might disseminate to distant organs (Escribano et al., 2017). Thus, in our study the genotype of *A. fumigatus* identified in non-sterile respiratory samples largely represented those found in deeper tissues. These results are in concordance with previous studies on fresh materials, in which *A. fumigatus* isolates obtained from deeper tissue were often identical to those from respiratory samples (de Valk et al., 2007).

A STR*Af* type from serum was only obtained from patient B. GM ratio in these serum samples was 4.0 and 5.6. In sera from patient A, C, and D GM ratios were <0.5 and STR*Af* typing was not successful. This finding suggests that successful STR*Af* typing in serum requires a certain threshold level of GM. A GM ratio threshold of >0.5 is one of the diagnostic criteria for classification of patients with IA (De Pauw et al., 2008; Boch et al., 2016; Eigl et al., 2017). Remarkable, only one out of four patients with proven IA exhibited a GM ratio of >0.5, suggesting that the sensitivity of this analysis in the diagnosis of IA is rather low. Indeed a recent study with twenty-six patients with proven/probable IA found that only 23% of patients exhibited serum GM ratio >0.5 (Boch et al., 2016). Then, we found that from sera with unsuccessful STR*Af* typing, four tested positive for *A. fumigatus* in the AsperGenius multiplex qPCR and three negative, demonstrating that in some situations STR*Af* typing is not successful despite the presence of *A. fumigatus* DNA. STR*Af* typing for these samples might be more successful if the multiplex PCR would be divided over monoplex PCRs, which normally enhances the individual STR*Af* signals. This was however not tested. Another possibility to improve STR*Af* typing from blood samples is the use of plasma instead of serum, as it was found that DNA concentrations are higher in plasma, leading to an increased sensitivity to detect *A. fumigatus* (White et al., 2015). Monoplex PCRs were also not performed for other samples, as only in one biopsy (C3) two dinucleotide repeats (2A and 2B) and one tetranucleotide repeat (4A) could not be amplified, while the six remaining loci yielded good interpretable peaks. These loci showed identical repeat numbers as many other cultured isolates of this patient. Therefore, these sample were considered to be identical.

Finally, using the AsperGenius multiplex qPCR assay we investigated the potential presence of azole resistance markers TR_34_, L98H, Y121F, and T289A in lung biopsies and serum samples positive for *A. fumigatus* (Chong et al., 2015). While amplification for most samples was achieved, it did not succeed for sample B19, although genotyping was successful. This likely represented a false negative result, possibly resulting from lower DNA quality. In samples with *A. fumigatus* no resistance mutations were identified. In sample B16 the TR_34_ melting curve did not correspond with wildtype or mutant control. This sample was not further investigated by sequence analysis, so the cause of this discrepancy remains unknown. Amplification of resistance targets was not successful in 7 of 52 PCR reactions and these materials originated from three sera and a lung biopsy. The other six lung biopsies with *A. fumigatus* were successfully characterized, demonstrating a success rate of 86% for FFPE tissues, which is very similar to the performance of this assay in non-FFPE clinical materials (White et al., 2017; Buil et al., 2018; Postina et al., 2018).

In conclusion, the STR*Af* assay allows simultaneous detection as well as high discriminatory genotyping of *A. fumigatus* directly in FFPE tissue samples without the need for culture. This approach allows (retrospective) examination of *A. fumigatus* in patients with IA and might be used toward a better understanding of the pathogenesis of this disease.

## Author contributions

TdG, FH, WV, PV, and JM acquisition, analysis and interpretation of the data. TdG and FH statistical analysis. TdG, FH, and JM drafted the manuscript. TdG, FH, WV, PV, AC, and JM reviewed and modified the manuscript.

### Conflict of interest statement

JM received grants from F2G and Merck. He has been a consultant to Scynexis and Merck and received speaker's fees from Merck, United Medical, TEVA and Gilead Sciences. PV received grants from Gilead Sciences, F2G, MSD, Thermo-Fisher and Pfizer. The remaining authors declare that the research was conducted in the absence of any commercial or financial relationships that could be construed as a potential conflict of interest.
